# Analysis of Major Bacteria and Diversity of Surface Soil to Discover Biomarkers Related to Soil Health

**DOI:** 10.3390/toxics10030117

**Published:** 2022-03-01

**Authors:** Heejung Kim, Yong-Ha Park, Jae E. Yang, Hyuck-Soo Kim, Sung-Chul Kim, Eun-Ji Oh, Jinah Moon, Wonsil Cho, Wonsik Shin, Chaerim Yu

**Affiliations:** 1 Department of Geology, College of Natural Sciences, Kangwon National University, Chuncheon 24341, Korea; hydroqueen@kangwon.ac.kr (H.K.); moon88@kangwon.ac.kr (J.M.); hydrocho@kangwon.ac.kr (W.C.); shinwg77@kangwon.ac.kr (W.S.); qqq0130@kangwon.ac.kr (C.Y.); 2 Korea Environment Institute, Sejong 30147, Korea; ejoh@kei.re.kr; 3 Department of Biological Environment, Kangwon National University, Chuncheon 24341, Korea; khs25@kangwon.ac.kr; 4 Department of Bio-Environmental Chemistry, Chungnam National University, Deajeon 34134, Korea; sckim@cnu.ac.kr

**Keywords:** soil health, surface soil, microbial community, biomarker, bacterial diversity

## Abstract

The discovery of biomarkers for assessing soil health requires the exploration of organisms that can explain the core functions of soil and identification of species with major roles in these functions. However, identifying specific keystone markers within the soil microbiota is challenging. Next-generation sequencing (NGS)-based molecular-biological methods have revealed information on soil biodiversity; however, whether this biodiversity is related to soil health remains unclear. In this study, we performed NGS on grassland surface soil to compare the prokaryotic and eukaryotic genetic diversity to determine the chemical soil quality and examined markers associated with soil health. Microorganisms associated with the nitrogen cycle, bioremediation, plant pathogenicity, antibiotic production, and material degradation showed potential for use as markers. To propose a framework for soil health assessment, we not only used traditional indicators, such as chemical and physical measures, but also assessed metagenomics data of soil by land use to identify the major factors influencing the microbial structure in soil. Moreover, major keystone species were identified. Furthermore, the microbial genetic diversity of generally healthy surface soil, such as forests, farmland, and parks, was determined. These findings provide basic data for exploring soil health-related biomarkers.

## 1. Introduction

Living organisms in soil, including the soil microbiota, play important roles in supporting life on earth. Climate changes and anthropogenic threats to soil such as intensive agriculture can greatly affect soil functions. Compared to soil indicators based on physical and chemical measures that can be used to assess soil quality, bioindicators remain controversial. Although these indicators typically have key functions and important regulatory roles (known as keystone species), identifying specific keystone markers within the vast functional redundancies of the soil microbiota remains challenging [1].

Next-generation sequencing (NGS)-based molecular techniques have been used to study soil microbial diversity and soil microorganisms useful for producing high-quality plants (agricultural soil environment and agriculture-related microorganisms). These studies reported the bacterial and fungal diversity according to the specific treatment of field soil; microbial diversity according to soil depth on a poplar farm; association between soil depth and native and exotic plant species; and comparison of the soil microbiota in natural and re-seeded grassland [2,3,4,5]. The results suggested that the soil depth is a major factor affecting the networking between the microbiota structure and abiotic factors, including interactions with fungi at approximately 1 m below the soil surface and microbial diversity at depths of 0–20 and ≥21 cm. The results also demonstrated that the surface soil has important effects on plant growth. 

Other studies suggested that the following groups of indicators can be used as indicators of the health and quality of soil based on NGS metadata: (i) Microorganisms beneficial to plants [nitrogen-fixing bacteria—symbiotic (*Rhizobia* etc.) or plant-associated bacteria (*Azospirillium* and *Paenibacillius* etc.); phosphate-solubilizing bacteria—*Pseudomonas* and Bacillus etc.; and bacteria inducing induced systemic resistance in plants and fungi forming beneficial symbionts with plants—arbuscular mycorrhizal and ectomycorrhizal fungi]; (ii) microorganisms harmful to plants (*Fusarium* genus is plant pathogen and related markers are assessed as factors negatively affecting plant growth); (iii) other potential genetic markers [anti-pathogen compounds that block pathogenic microorganisms and indole acetic acid which promotes plant growth via production of plant growth hormones]; and (iv) soil microorganisms related to nutrient cycling [nitrogen fixation (*nifH*), nitrification (*amoA*), denitrification (*nir*, *nor*), N immobilization (glutamine synthase-encoding gene), N mineralization (protease-encoding genes), organic C mineralization (β-glucosidase-encoding genes), carbon dioxide fixation (RUBISCO-encoding genes), and organic P mineralization (acid and alkaline phosphomonoesterase)] [6]. Other ecological services performed by the soil microbiota include regulation of biogeochemical cycles, retention and delivery of nutrients to primary producers, maintenance of soil structure and fertility, bioremediation of contaminants, supply of clean drinking water, flood and drought mitigation, erosion control, regulation of atmospheric trace gases, pest and pathogen control, and regulation of plant production through secondary metabolites (non-nutritive biochemical substances).

Specific genes, taxa, or groups with principles based on such functionalities may be useful as indicators [7]. However, some microorganisms may appear at different times and locations and may vary in the presence of different plant species. For example, when *Carex arenaria* was cultivated in ten different types of soil, the diversity of rhizobacteria was more similar to that in bulk soil compared to the diversity of rhizobacteria in other soil types [8,9]. Examining the key functions based on soil microorganism metadata may lead to the identification of markers in multi-function soil beyond the single-microorganism level (co-occurrence of specific microbial taxa), enabling the use of network connectivity as an indicator of soil health. Moreover, sampling at different times and locations may be more important than assessing all DNA from the soil microbiota. To develop a framework for soil health assessment, we used traditional indicators, such as chemical and physical measures, as well as molecular-biological metagenomics data of soil by land use to identify the major factors influencing the microbial structure in soil. Moreover, we identified major keystone species according to land use. 

## 2. Materials and Methods

### 2.1. Sampling

**Figure 1:**
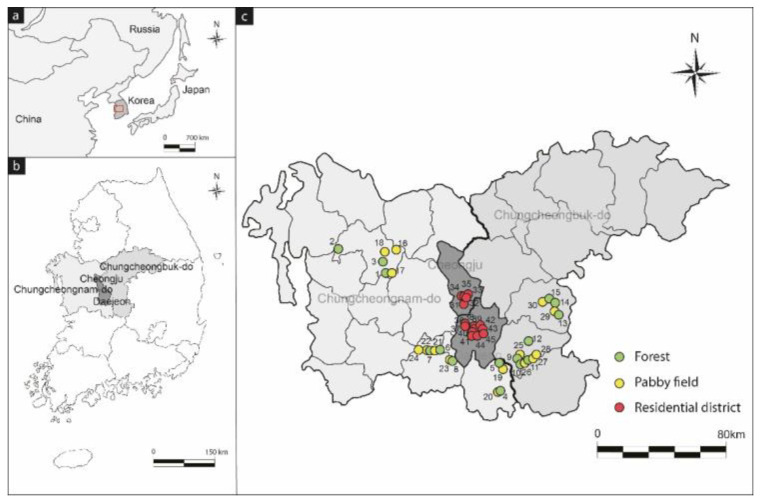
Locations and study area (**a**,**b**) and sampling points (**c**).

The study area was comprised of forests, agricultural lands, and residential districts. Forty-five samples were collected in 15 samples of the forest and paddy soil in Chungcheong province in southwest Korea (Yesan, Geumsan, Gongju, Okcheon, and Boeun) and 15 samples from residential districts in Sejong and Daejeon (Figure 1). 

### 2.2. Physico-Chemical Parameter Analysis

Soil sampling and analysis were performed according to the guidelines of the National Academy of Agricultural Science in Korea (NAAS, 2010) [10]. The collected soil samples were filtered through a 2-mm (10 mesh) sieve after air-drying. The soil texture was determined using the micro-pipette method [11]. The pH and electrical conductivity of the samples were determined for a 1:5 soil:water (*w*/*v*) suspension using a pH meter and conductivity meter (MP220, Mettler Toledo, Columbus, OH, USA) [4]. The cation exchange capacity was analyzed using the 1 M CH_3_COOH extraction method [2]. The soil organic content was measured as described by Walkley and Black. Effective phosphoric acid was determined using the Bray No.1 method with molybdenum blue dye and measured on a UV spectrophotometer (UV-1800, Shimadzu, Kyoto, Japan) [2]. The inorganic nitrogen content, as NH_4_-N and NO_3_-N, was determined using a QuikChem automated ion analyzer (QuikChem 6000 Series, Lachat Instruments, Milwaukee, WI, USA), after extraction with 2 M KCl [3].

### 2.3. Soil Enzyme Analysis

Activity assays of the individual soil enzymes β-glucosidase, Urease, acid phosphomonoesterase (also known as acid phosphatase), and arylsulfatase were performed as described by Acosta-Martínez et al. (2018) [5] and according to other previous studies [6,7,8,9,10,11,12]. To analyze β-glucosidase, 0.5 g of fresh soil was incubated at 30 °C for 1 h to convert *p*-nitrophenyl-β-glucoside into *p*-nitrophenol, and absorbance was measured with a UV/vis spectrometer at 400 nm (Evolution 60S, Thermo Fisher Scientific, Waltham, MA, USA). 

For phosphatase analysis, 1 g of fresh soil was incubated for 1 h at 37 °C after adding 0.2 mL toluene, 0.025 M *p*-nitrophenyl phosphate, and 1 mL of modified universal buffer (pH 6.5) to the test tube. Next, 4.0 mL of 0.5 M NaOH and 1 mL of 0.5 M CaCl_2_ were added to quench the reaction. The filtrate was measured with a UV/vis spectrometer at 400 nm. A calibration curve for both β-glucosidase and phosphatase was generated with 0.1 M Tris buffer mixed with 0.4–1.7 µg *p*-nitrophenol. Soil enzyme activity was expressed as µg *p*-nitrolphenol produced by 1 g dry weight soil/h (Table 1). 

### 2.4. NGS Analysis

DNA was extracted using a DNeasyPowerSoil Kit (Qiagen, Hilden, Germany) according to the manufacturer’s instructions. Sequencing libraries were prepared according to the Illumina 16S Metagenomic Sequencing Library protocols to amplify the V3 and V4 region. The input genomic DNA (2 ng) was PCR-amplified in 5× reaction buffer, 1 mM of dNTP mix, 500 nM each universal F/R PCR primer, and Herculase II fusion DNA polymerase (Agilent Technologies, Santa Clara, CA, USA). The cycling conditions for the first round of PCR were as follows: 3 min at 95 °C for denaturation, and 25 cycles of 30 s at 95 °C, 30 s at 55 °C, and 30 s at 72 °C, followed by a 5-min final extension at 72 °C. The universal primer pair with Illumina adapter overhang sequences used for the first amplifications are as follows: V3-F: 5′-TCG TCG GCA GCG TCA GAT GTG TAT AAG AGA CAG CCT ACG GGN GGC WGC AG-3′, V3-F: 5′-TCG TCG GCA GCG TCA GAT GTG TAT AAG AGA CAG CCT ACG GGN GGC WGC AG-3′, V4-R: 5′-GTC TCG TGG GCT CGG AGA TGT GTA TAA GAG ACA GGA CTA CHV GGG TAT CTA ATCC-3′.

Illumina MiSeq sequencing was conducted to analyze the bacterial community structures. Finally, All samples were analyzed using Illumina Miseq sequencing (San Diego, CA, USA) by Macrogen, Inc. (Seoul, Korea). Quantitative Insights into Molecular Ecology (QIME) software was used for comparisons at the phylum to species levels through data trimming and analyzing the alpha diversity [13]. Sequence reads were analyzed using QIIME software version. Ambiguous and chimeric sequences were removed, and sequences were classified into operational taxonomic units (OTUs) at 97% similarity using the CD-HIT-OTU program (Macrogen, Inc., Seoul, Korea). The taxonomy of each OTU was assigned based on the NCBI 16S microbial database. Shannon’s diversity index, Simpson’s diversity index, Chao 1 richness, and Ace richness were calculated in QIIME and used to compare the soil fungal alpha diversity. Venn diagrams of unique and shared OTUs were drawn to highlight the similarities and shared sequences between the different samples.

### 2.5. Nonmetric Multidimensional Scaling and Canonical Correspondence Analysis

Nonmetric multidimensional (NMDS) analysis was performed to determine the patterns of similarity (Bray-Curtis similarity) in the structure of the microbial community between treatments [14]. Canonical correspondence analysis (CCA) was conducted to explore the association of the microbial community composition with the soil characteristics. NMDS analysis and CCA were performed using the “vegan” package in R version 3.2.0 for Windows (The R Project for Statistical Computing, Vienna, Austria). MDS, which is used to express similarity/dissimilarity between samples by plotting points in two- or three-dimensional space, is a statistical method for identifying patterns or structures inherent within data through visualization of the proximity between samples. This approach is applied in various fields, including the social and natural sciences, to analyze the similarity/dissimilarity in microbial communities that change according to environmental factors. In MDS, the distance between samples is calculated using the Euclidean distance matrix. In contrast, NMDS is used when data are given on an order scale. When the distance between samples is given in order, the distance is generated by converting the order scale to be the same as the attributes of distance.

## 3. Results and Discussion

### 3.1. Alpha Diversity Index with Different Land Use Types

We analyzed 247,192 reads from 15 agriculture samples and 259,856 reads from 15 forest samples. Chao1 and Shannon’s index were high in the agriculture samples, indicating richer microbial diversity in agriculture soil than in forest soil. The inverse Simpson and Good’s coverage values are indicators of the similarity of microbiome diversity within the range of soil samples. The sequencing coverage was similar between groups (Table 2). 

### 3.2. Phylum-Level Analysis

**Figure 2:**
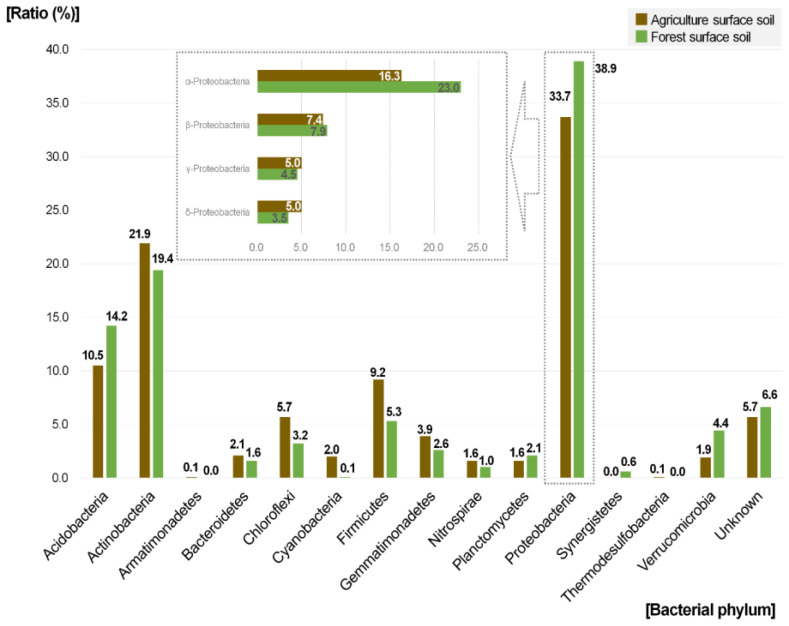
Comparison of bacterial diversity between agricultural and forest land use types at the phylum levels.

As shown in Figure 2, Proteobacteria was the dominant phylum in both agriculture and forest soil samples, followed by Actinobacteria and Acidobacteria. The phyla Firmicutes, Actinobacteria, and Chloroflexi showed relatively higher levels in agriculture soil than in forest soil, with differences of 3.9%, 2.5%, and 2.5%, respectively. In contrast, the phyla Proteobacteria, Acidobacteria, and Verrucomicrobia showed higher levels in forest soil than in agriculture soil, with differences of 5.2%, 3.7%, and 2.5%, respectively. The findings were similar to those reported by Lee et al. (2021) [13]. However, unlike previous studies showing that Acidobacteria was dominant, this phylum showed approximately 3.7% higher levels in forest soil than in agriculture soil in the present study. Acidobacteria may have been affected by factors other than the type of land use, such as regional differences. 

In agriculture soil, the phyla Cyanobacteria, Nitrospirae, Bacteroidetes, Gemmatimonadetes, Firmicutes, and Chloroflexi and class Gammaproteobacteria were relatively dominant. In forest soil, the phyla Synergistetes, Planctomycetes, Verrucomicrobia, and Acidobacteria and class Alphaproteobacteria were relatively dominant. The phylum Proteobacteria, which showed a relatively lower level in agriculture soil, and Firmicutes [9.2% with ≥3.9% difference relative to other use (forest 5.3%)], Cyanobacteria [2.0% with ≥1.9% difference relative to other use (forest 0.1%)], Gemmatimonadetes [3.9% with ≥1.3% difference relative to other use (forest 2.6%)], Chloroflexi [5.7% with ≥2.5% difference relative to other use (forest 3.2%)], and Nitrospirae [1.6% with ≥0.6% difference relative to other use (forest 1.0%)], which showed relatively higher levels than in other use land, showed characteristic differences at the phylum level according to the land use type. Additionally, the class Alphaproteobacteria [23.0% with ≥6.7%], Acidobacteria [14.2% with ≥3.7%], Verrucomicrobia [4.4% with ≥2.5%], and Synergistetes [0.6% with ≥0.4%] showed relatively higher levels in forest soil than in other land use types.

Agriculture soil contained 28, 15, and 2 OTUs at ratios of 0.5–0.9%, 1.0–2.9%, and ≥3%, respectively, for a total of 45 OTUs with a ratio ≥ 0.5%. Forest soil contained 30, 12, and 5 OTUs with ratios of 0.5–0.9%, 1.0–2.9%, and ≥3%, respectively, for a total of 47 OTUs with a ratio ≥ 0.5%, showing similar results. In contrast, there were 295 agriculture soil OTUs with a ratio of ≥0.1%, including others, which was higher than the 242 OTUs detected in forest soil. Particularly, OTUs with a ratio ≥ 0.1% but <0.5% were abundant in agriculture soil. Moreover, 103 and 50 OTUs appeared in agriculture and forest soil, respectively, indicating that agriculture soil has greater diversity than forest soil, with many more OTUs characteristically present in agriculture soil (Table 3).

The major characteristics of 103 OTUs that appeared as codominant species in agriculture soil were determined (Table 3). Of the nine species within the *Nocardioides* genus, seven species were present only in agriculture soil. After excluding species with a difference of ≥0.5% as compared to forest soil and those found in residential (park) soil in the present study and species that appeared in soil other than agriculture soil in previous studies, there were 10 main species (*Bacillus cucumis*, *Hydrogenispora ethanolica*, *Luteitalea pratensis*, *Micromonospora oryzae*, *Nitrospira moscoviensis*, *Nocardioides mesophilus*, *Paeniglutamicibacter cryotolerans*, *Sphaerobacter thermophilus*, *Tepidimonas taiwanensis*, and *Terrabacter carboxydivorans*). These species were mostly isolated from environments such as soil around rhizosphere. Actinomycetales, high temperature, and functionalities (mainly beneficial effects such as ethanol and hydrogen production, urea decomposition, carbon monoxide oxidation, etc.) were reported [15]. Other species that appeared as codominant species in agriculture soil compared to in forest soil (≥0.5% difference) were as follows: *Sphingomonas limnosediminicola* (2.2% difference), *Bacillus zanthoxyli* (1.4% difference), *Streptomyces gilvifuscus* (0.8% difference), *H. ethanolica* (0.7% difference), *T. taiwanensis* (0.7% difference), *Vicinamibacter silvestris* (≥0.5% difference), *Dehalogenimonas alkenigignens* (≥0.5% difference), and *L. pratensis* (≥0.5% difference). 

The major characteristics of the 50 species that were codominant species in forest soil were as follows. Of the seven species within the *Paraburkholderia* genus, six species were present only in forest soil. After excluding species with a difference of ≥0.5% as compared to in agriculture soil and found in residential (park) soil as well as species that appeared in soil other than agriculture soil in previous studies, there were six species (*Actinomadura rifamycini*, *Azospirillum agricola*, *Gelria glutamica*, *Methylobacillus flagellatus*, *Terriglobus saanensis*, and *Thermanaerovibrio velox*). These species were isolated from soil or other sample types and predicted to be associated with the nitrogen cycle or groundwater contamination. Other species found to be codominant species in forest soil but not in agriculture soil (≥0.5% difference) were as follows: *Acidobacterium ailaaui* (−2.2% difference), *Chthoniobacter flavus* (−2.2% difference), *Bradyrhizobium namibiense* (−2.1% difference), *Actinoallomurus vinaceus* (−1.7% difference), *Pseudolabrys taiwanensis* (−1.6% difference), *Paludibaculum fermentans* (−1.6% difference), *Natranaerobaculum magadiense* (−1.3% difference), *Rhodoplanes tepidamans* (−1.0% difference), *Acidibrevibacterium fodinaquatile (*−0.8% difference), *Aciditerrimonas ferrireducens* (−0.6% difference), *Aliidongia dinghuensis* (−0.6% difference), *Edaphobacter modestus* (−0.6% difference), *Methylophilus methylotrophus* (−0.6% difference), *Mycolicibacterium mucogenicum* (−0.5% difference), and *Planctopirus limnophila* (−0.5% difference) (Table 4). 

**Figure 3:**
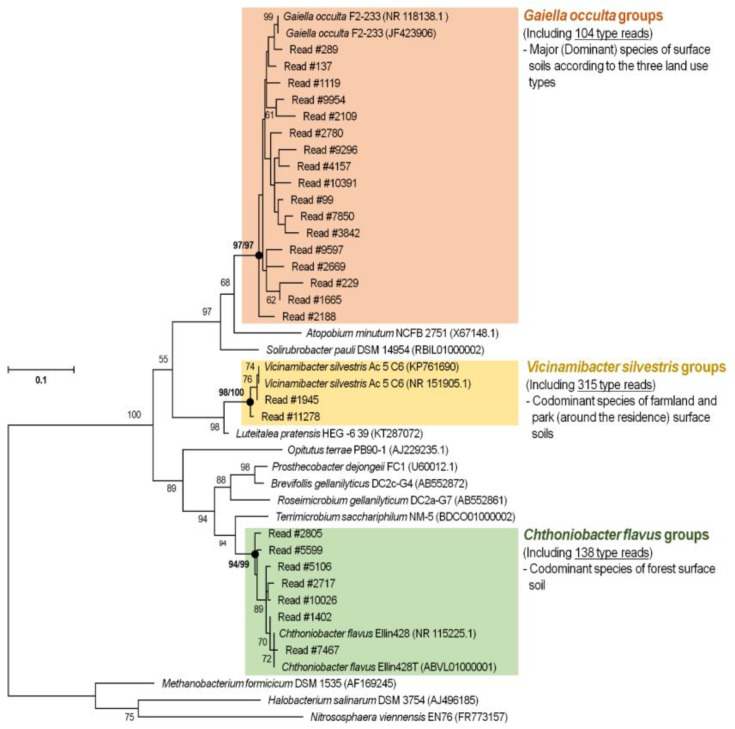
Maximum likelihood (using the Tamura-Nei distance model) tree showing the phylogenetic position of major and codominant species from topsoil of three land use types based on 16S rRNA gene sequences. Closed circle indicates corresponding branches recovered in the maximum likelihood/neighbor-joining (using the Kimura 2-parameter distance model) trees. Bootstrap values less than 50% based on 1000 replications are shown. Three archaea [*Methanobacterium formicicum* DSM 1535 (AF169245), *Halobacterium salinarum* DSM 3754 (AJ496185), and *Nitrososphaera viennensis* EN76 (FR773157)] were used as an out group. Bar, 0.1 nucleotide substitutions.

*Gaiella occulta* was the dominant species in both agriculture and forest soil, whereas *V. silvestris* was the subdominant species in agriculture and residential (park) soil and *C. flavus* was the subdominant species in forest soil. *Gaiella occulta*, which was dominant in all types of soil, appeared at a rate of 4.0% in agriculture soil and 4.1% in forest soil, with 104 types of OTUs (Figure 3). *Vicinamibacter silvestris*, which was subdominant in agriculture and residential (park) soil, showed a prevalence of 3.7% (3.2% in forest soil for top 5 OTUs among species excluding others) and there were 315 types of OTUs. Moreover, *C. flavus*, which was subdominant in forest soil, showed a rate of 3.5% [1.3% in agriculture for top 10 OTUs among species excluding others] and there were 138 types of OTUs. The dominant species *G*. *occulta* was isolated from aquifer mineral water by Albuquerque et al. (2011) [32] and has been reported to be related to nucleic acids (clones) reported in soil and lakes. *Vicinamibacter silvestris* was isolated and reported in semi-arid soil from subtropical savanna region by Huber et al. (2016) [33], whereas *C*. *flavus* was isolated and reported in soil from Australian rye fields and clover pasture by Sangwan et al. (2004) [34]. The dominant and subdominant species may be associated with the environment and plants. Moreover, by examining the characteristics of the dominant microorganisms and bacteria diversity, this information can be used as a major indicator of soil health. However, there are disadvantages to NGS-based research results, and it is considered necessary to perform culture-based follow-up studies.

### 3.3. NMDS and CCA 

NMDS and metagenomic patterns of microorganisms by land use were analyzed for CCA between the major dominant species and physicochemical factors. 

**Figure 4:**
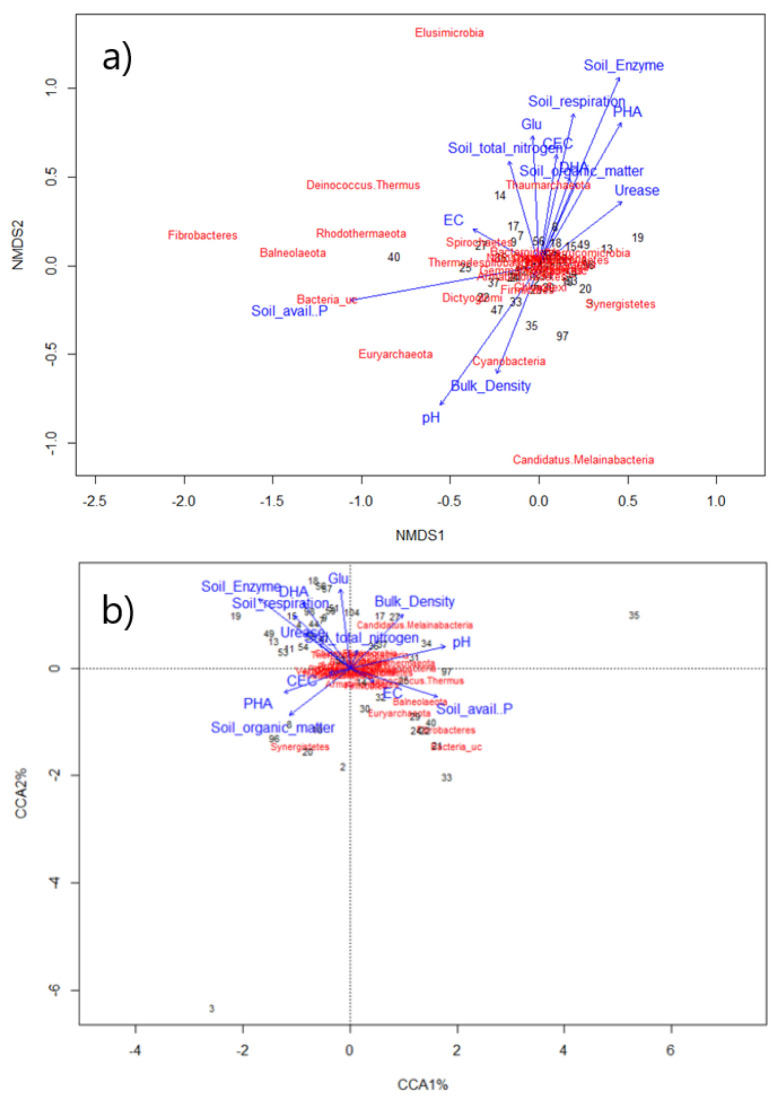
Results of (**a**) non-metric multidimensional scaling (NMDS) and (**b**) canonical correspondence analyses (CCA) for bacteria in the 30 soil samples with physicochemical data.

To increase the accuracy of the relative distance, fitness is expressed as stress values. pH, soil organic matter, soil available phosphorous, soil enzyme, and PHA showed high correlation coefficients with the microbial results by land use. Similar results were found using CCA. Particularly, pH showed major parameter with microorganisms by land use, with high correlation coefficients in NMDS analysis and CCA. These results indicate that microorganisms affecting soil health are closely associated with pH (Figure 4).

## 4. Conclusions

To propose a framework construct for soil health assessment, we used not only traditional indicators, such as chemical and physical measures, but also molecular-biological metagenomics data of soil by land use to assess the major factors influencing the microbial structure in soil. We observed close associations in the order of PHA, pH, phosphorous, and soil enzyme among the major indicators for assessment of soil health. Soil organic matter and pH were also significant factors. pH was found to have a major influence on soil health and major microbial communities. The pH and soil microorganism data can be used to maintain and manage soil health. Furthermore, metadata on various soil microorganisms should be collected continuously to define markers of multi-functions in soil. 

## Figures and Tables

**Table 1:** Characteristics of surface soil with agriculture and forest as land use types.

Characteristic	Land Use Types of Surface Soil		Evaluation Criteria ^a^
	Agriculture (*n* = 15)	Forest (*n* = 15)	
Soil texture (%)			
Clay	17.40	20.13	-
Silt	17.13	19.67	
Sand	65.47	60.20	
pH	5.84	5.12	5.5–6.5
Electrical conductivity (dS/m)	0.64	0.61	<1.0 dS/m
Soil organic matter [g/kg (%)]	34.12 (3.41)	54.52 (5.45)	20.0–30.0 g/kg (or >3.0%)
Total nitrogen [g/kg (%)]	1.57 (0.16)	1.85 (0.18)	Forest (>0.25%)
Available phosphorus (mg/kg)	436.15	157.06	
Exchange capacity (cmol/kg)			
Ca	5.74	5.95	
K	0.19	0.22	
Mg	1.06	1.10	
Na	0.02	0.03	Forest (0.15–0.5 cmol/kg)
Cation exchange capacity (cmol/kg)	10.66	13.60	
Physical properties			
Soil moisture (%)	16.46	18.95	-
Bulk density (g/cm^3^)	1.40	1.20	-
Porosity (%)	47.25	54.68	-
Soil aggregate formation (mm)	0.42	0.59	-
Soil respiration [CO_2_ (mg/kg; day)]	62.15	90.02	-
Soil enzyme and activity			
arylsulfatase (*p*-nitrophenol µmol/h/g)	4.42	14.32	-
Dehydrogenase (TPF µg/g)	11.32	21.52	-
β-Glucosidase (*p*-nitrophenol µmol/h/g)	2.72	4.88	-
Urease (µg-N/g/2 h)	13.03	21.34	-
Cultivable microorganisms (CFU, ×10^6^/g)	6.50	19.53	-

^a^ Available phosphorus, paddies and/or forest (80–120) and field (300–550); exchange capacity (Exch.)-Ca, agriculture (5.0–6.0) and forest (0.25–5.0); exch.-K, agriculture [field (0.5–0.8) and paddy (0.2–0.3)] and forest (0.25–5.0); exch.-Mg, agriculture (1.5–2.0) and forest (>1.5); exch.-Na, agriculture (1.5–2.0) and forest (>1.5); CEC, agriculture (>14.0) and forest (12.0–20.0).

**Table 2:** Alpha diversity index of agriculture and forest soils.

Alpha Diversity	Land Use	
	Agriculture	Forest
Chao 1	1356.88–2447.70 (2089.56)	915.22–2023.01 (1509.48)
Shannon	8.35–9.33 (8.93)	6.99–9.01 (8.35)
Inverse Simpson	0.99–1.00 (0.99)	0.98–1.00 (0.99)
Good’s Coverage	0.93–0.99 (0.97)	0.97–0.99 (0.98)

Average values are shown in brackets.

**Table 3:** Operational taxonomic units (OTUs) of ratio (%) of agriculture land and forest soils.

Land Use	OTUs	Ratio (%) of OTUs (Others Except)				OTUs	
		0.5–0.9	1.0–2.9	>3.0	Total	Agricultural Land	Forest
Agricultural land	295	28	15	2	45	103	-
Forest	242	30	12	5	47	-	50

**Table 4:** Dominating species in surface soil in agriculture and forest land use types.

Land Use	Major Species	Ratio (%)	Characteristics	Reference
Agriculture	*Bacillus cucumis*	0.6	Gram-positive-staining, aerobic, endospore-forming bacterial strain, isolated from the stem of a cucumber plant, studied in detail for its taxonomic position.	Kämpfer et al., 2016 [16]
	*Hydrogenispora ethanolica*	0.7	Anaerobic, spore-forming, ethanol-hydrogen-coproducing bacterium, designated LX-BT, isolated from an anaerobic sludge treating herbicide wastewater.	Liu et al., 2014 [17]
	*Luteitalea pratensis*	0.5	Novel representative of Acidobacteria subdivision 6 isolated from grassland soil in Thuringia, Germany.	Vieira et al., 2017 [18]
	*Micromonospora oryzae*	0.6	Actinomycete strain isolated from root internal tissues of upland rice (*Oryza sativa*).	Kittiwongwattana et al., 2015 [19]
	*Nitrospira moscoviensis*	0.8	Gram-negative, non-motile, non-marine, nitrite-oxidizing bacterium was isolated from an enrichment culture initiated with a sample from a partially corroded area of an iron pipe of a heating system in Moscow, Russia.	Ehrich et al., 1995 [20]
Agriculture	*Nocardioides mesophilus*	0.5	Short coccoid- to rod-shaped, motile, mesophilic actinobacterium, strain MSL-22(T), isolated from soil on Bigeum Island, Korea.	Dastager et al., 2010 [21]
	*Paeniglutamicibacter cryotolerans*	0.7	Two novel cold-tolerant, Gram-stain-positive, motile, facultatively anaerobic bacterial strains, LI2(T) and LI3(T), isolated from moss-covered soil from Livingston Island, Antarctica, near the Bulgarian station St. Kliment Ohridski. A rod-coccus cycle was observed for both strains.	Ganzert et al., 2011 [22]
	*Sphaerobacter thermophilus*	1.0	Phenotypic and genotypic properties of a Gram-positive non-spore-forming strain belonging to the dominant flora grown on aerobe-thermophilically treated sewage sludge.	Demharter et al., 1989 [23]
	*Terrabacter carboxydivorans*	0.7	Bacterial strain, PY2(T), capable of oxidizing carbon monoxide, isolated from a soil sample collected from a roadside at Yonsei University, Seoul, Korea.	Kim et al., 2011 [24]
	*Tepidimonas taiwanensis*	0.8	Bacterial strain designated I1-1(T) isolated from a hot spring located in the Pingtung area, southern Taiwan.	Chen et al., 2006 [25]
Forest	*Azospirillum agricola*	−0.8	Polyphasic approach was used to characterize a novel nitrogen-fixing bacterial strain, designated CC-HIH038T, isolated from cultivated soil in Taiwan.	Lin et al., 2016 [26]
	*Actinomadura rifamycini*	−0.5	Gram-reaction-positive aerobic actinomycete, designated as strain IM17-1(T), isolated from a honey bee (*Apis mellifera*) hive in Chiang Mai Province, Thailand.	Promnuan et al., 2011 [27]
	*Gelria glutamica*	−0.5	Novel anaerobic, Gram-positive, thermophilic, spore-forming, obligately syntrophic, glutamate-degrading bacterium, strain TGO(T), isolated from a propionate-oxidizing methanogenic enrichment culture.	Plugge et al., 2002 [28]
Forest	*Methylobacillus flagellatus*	−0.7	New methyltrophic bacterium which utilizes methanol as a sole source of carbon and energy isolated from soil. It was a Gram-negative, nonmotile, nonspore-forming rod, and strictly aerobic bacterium. Catalase and oxidase activities were present.	Govorukhina et al., 1997 [29]
	*Terriglobus saanensis*	−0.6	Two aerobic bacterial strains, designated as SP1PR4(T) and SP1PR5, isolated from tundra soil samples collected from Saana fjeld, north-western Finland.	Männistö et al., 2011 [30]
	*Thermanaerovibrio velox*	−0.6	Moderately thermophilic, organotrophic bacterium with vibrioid cells isolated from a sample of a cyanobacterial mat from caldera Uzon, Kamchatka, Russia, and designated strain Z-9701T.	Zavarzina et al., 2000 [31]

## Data Availability

Not applicable.
